# Miscellaneous Notices

**Published:** 1846-06

**Authors:** 


### Miscellaneous Notices.

### AMERICAN SOCIETY OF DENTAL SURGEONS.

As this is the last number of the Journal that will be issued previously to the seventh annual meeting of the American Society of Dental Surgeons, we cannot let the opportunity pass, without urging upon the members the importance of being present on that occasion. The meeting will be one of peculiar interest, as business of importance, growing out of the proceedings of the last meeting, will be transacted. It is desirable, therefore, that the attendance of the members should be as general as possible, and, although the sacrifice of time and money, which the more distant members may find it necessary to make, may be considerable, we believe all will be amply compensated in gratification as well as real benefit derived from the interchange of professional views. The meetings heretofore have been well attended, and we hope to see more at the next than have been present at any of the preceding ones, and lest any of the members should fail to receive the notice which it is the duty of the recording secretary to send to each, specifying the time and place at which it is to be held, we would remind all such, should there be any, that it will commence the first Tuesday in August, in New York.

The Society has now been in existence six years, and numbers among its members many of the most talented men and skilful practitioners in the dental profession, both in Europe and America. Indeed, the conditions of membership are such, that none but men of respectable professional attainments can obtain admission. This is as it should be, and if others have at any time been elected to membership, and that some have is very probable, the precautions at present observed by the Society in the admission of applicants, is such as to preclude them for the future. In settling the conditions of membership, the Society, while it has thrown open wide the door of admission to such as are really worthy, has found it necessary to exact the most indisputable evidences of professional ability and moral rectitude, before permitting any to enter, as may be seen by an examination of its constitution and by-laws, published in the fifth volume of the Journal. The character and usefulness of the Society depend upon the strict enforcement of its regulations in regard to this matter.

The beneficial effects, both to the profession and community at large, which have resulted from the formation and existence of this association, are so striking and manifest, as to have been observed and felt, not only throughout the length and breadth of this, but also in other countries. It, in connection with other instrumentalities, and to most of which it has given rise, has given a character and respectability to the profession it never before enjoyed. It has contributed in an eminent degree to elevate the standard of professional acquirements, as well as to the general adoption of a more scientific and uniform system of practice. It has also awakened a spirit of inquiry and emulation among the widely scattered members of the profession, and sustained a medium of communication through which all new theories and improvements in practice have been made known to such as have been desirous of keeping pace with the progress of the science and art of dental surgery.

But, as useful as the Society has already been, in establishing a uniform and correct system of practice among its members, and in the dissemination of a knowledge of every thing valuable to the dental branch of medicine, it is destined, if prudently and properly conducted, to be still more useful, as it will become the depository of the accumulated experience and practical improvements and discoveries of its members. Membership, therefore, in this Society, should not be lightly prized, but no one should be satisfied with this, without the benefit, to say nothing of gratification, to be derived, of attendance on its annual meetings. There are some who have been members for four, five and even six years, who have not as yet attended a single meeting. We should be glad to see these, as well as those whom we have been accustomed to meet regularly, at the next meeting. We hope they will make it a point to attend, believing they will not regret the expense and loss of time which they may have to incur in doing so.

*Balt. Ed.*

---

*Surgeon Dentists in Paris.*—By a recent decision of the law courts in Paris, it appears that surgeon dentists are not qualified to practice their branch of the profession in France, unless possessed of the diploma of M. D., or at least of that of *officiér dé santé.* Mr. Rogers, an Englishman, was fined twenty-five francs, by the *tribunal* correctionelle, for so practicing without a medical qualification, and the *Cour Royale,* after hearing his appeal, decided against him, confirming the previous decision, and cast him in costs.

---

*Salivary Calculus.*—The largest accumulation of this substance which we have ever seen upon a single tooth, was presented to us by Dr. Wilkes Allen, of Boston, a graduate of the Baltimore College of Dental Surgery. Its longest diameter is one inch and one-eighth, and its shortest seven-eighths, and it is nearly five-eighths of an inch thick. It is of an irregular oval shape, quite smooth, except on its inner surface, where it had rested against the gum. Embedded in its substance is the entire crown and neck of an inferior dens sapientiæ, the tooth upon which it was deposited, and which, in the removal of this, was brought away with it. It is of a light brown color, indicative of a bilious temperament, and weighs two drachms and seventeen grains.

*Balt. Ed.*

We are also indebted to Dr. E. Noyes, of Baltimore, for another very large specimen of this substance, which had been deposited on an inferior incisor.—*Balt. Ed.*

---

*Singular Deviation in the Growth of a Supernumerary Tooth.*—Some very remarkable deviations in the growth of human teeth are recorded by Albinus, Hunter, Waite and other writers, but the most singular one which has ever fallen under our observation, is exhibited in the upper jaw of an adult skull in the museum of the Baltimore College of Dental Surgery. The natural teeth are all well formed and well arranged, but between the extremities of the roots of the upper incisors, in the substance of the jaw, there is a supernumerary tooth, the crown of which points upwards towards the crest of the nasal plates of these bones. The whole tooth is about one inch in length, and the coronal extremity is nearly on a level with the floor of the nasal cavities.
*Balt. Ed.*

---

*To Delinquents.*—It is both disagreeable and troublesome to be under the necessity of calling on delinquents for the amount of their subscriptions, and yet we must either do so, or involve the American Society of Dental Surgeons in debt, or let our printer go unpaid. In the March number of the present volume of the Journal, we sent bills to most of our delinquent subscribers, requesting immediate payment. To this call some responded promptly, but it has been wholly disregarded by the greater number of those who were in arrears. We hope, however, they will not delay the payment of their subscriptions any longer, as we need the money, and their non-compliance with the terms of subscription not only occasions us great inconvenience, but causes us much labor which might otherwise be avoided. We do not believe the delinquency in a single case results from inability, but from thoughtlessness and neglect. We shall expect to hear from all such, immediately after they shall have received the present number of the Journal.

